# Limited Impact of 2 g/day Omega-3 Fatty Acid Ethyl Esters (Omacor^®^) on Plasma Lipids and Inflammatory Markers in Patients Awaiting Carotid Endarterectomy

**DOI:** 10.3390/md11093569

**Published:** 2013-09-20

**Authors:** Hayati M. Yusof, Abbie L. Cawood, Ren Ding, Jennifer A. Williams, Frances L. Napper, Clifford P. Shearman, Robert F. Grimble, Simon P.K. Payne, Philip C. Calder

**Affiliations:** 1 Faculty of Medicine, University of Southampton, Southampton SO16 6YD, UK; E-Mails: abbie.cawood@nutricia.com (A.L.C.); rending03@yahoo.co.uk (R.D.); jenny.williams2@uhs.nhs.uk (J.A.W.); flnapper@hotmail.co.uk (F.L.N.); c.p.shearman@soton.ac.uk (C.P.S.); r.f.grimble@soton.ac.uk (R.F.G.); P.C.Calder@soton.ac.uk (P.C.C.); 2 Department of Food Science, Universiti Malaysia Terengganu, 21030 Kuala Terengganu, Malaysia; 3 Department of Vascular Surgery, Queen Alexandra Hospital, Portsmouth PO6 3LY, UK; E-Mail: simon.payne@porthosp.nhs.uk; 4 National Institute for Health Research Southampton Biomedical Research Centre, University of Southampton and University Hospital Southampton NHS Foundation Trust, Southampton SO16 6YD, UK

**Keywords:** omega-3, fish oil, cytokine, adhesion molecule, cardiovascular disease

## Abstract

The objective of this study was to determine the effects of prescription omega-3 (*n-3*) fatty acid ethyl esters (Omacor^®^) on blood pressure, plasma lipids, and inflammatory marker concentrations in patients awaiting carotid endarterectomy. Patients awaiting carotid endarterectomy (*n* = 121) were randomised to Omacor^®^ or olive oil as placebo (2 g/day) until surgery (median 21 days). Blood pressure, plasma lipids, and plasma inflammatory markers were determined. There were significant decreases in systolic and diastolic blood pressure and in plasma triglyceride, total cholesterol, low density lipoprotein-cholesterol, soluble vascular cellular adhesion molecule 1, and matrix metalloproteinase 2 concentrations, in both groups. The extent of triglyceride lowering was greater with Omacor^®^ (25%) compared with placebo (9%). Soluble E-selectin concentration was significantly decreased in the Omacor^®^ group but increased in the placebo group. At the end of the supplementation period there were no differences in blood pressure or in plasma lipid and inflammatory marker concentrations between the two groups. It is concluded that Omacor^®^ given at 2 g/day for an average of 21 days to patients with advanced carotid atherosclerosis lowers triglycerides and soluble E-selectin concentrations, but has limited broad impact on the plasma lipid profile or on inflammatory markers. This may be because the duration of intervention was too short or the dose of *n*-3 fatty acids was too low.

Abbreviations

ACE, angiotensin-converting-enzyme; ARA, arachidonic acid; BMI, Body mass index; CRP, C-reactive protein; CVD, cardiovascular disease; DHA, docosahexaenoic acid; EPA, eicosapentaenoic acid; HDL, high density lipoprotein; IL, interleukin; IP, interferon gamma induced protein; LC, long chain; LDL, low density lipoprotein; MIG, monokine induced by gamma-interferon; MMP, matrix metalloproteinase; PUFA, polyunsaturated fatty acid; sCD40L, soluble CD40 ligand; sE, soluble endothelial; sICAM, soluble intercellular adhesion molecule; sVCAM, soluble vascular cellular adhesion molecule; TAG, triglyceride; TGF, transforming growth factor.

## 1. Introduction

Consumption of fish, especially oily fish, protects against cardiovascular disease (CVD) morbidity and mortality [1,2,3]. The effect of fish is believed to be mainly due to its component long chain omega-3 (*n*-3) polyunsaturated fatty acids (LC *n*-3 PUFAs) [3,4]. Indeed, in accordance with this, higher intake or status of LC *n*-3 PUFAs are also associated with lower risk CVD morbidity and mortality [3,4,5,6]. LC *n*-3 PUFAs act through modification of the CVD risk factor profile including blood pressure [7,8], plasma triglyceride (TAG) concentrations [9,10] and inflammation [11,12], amongst others [3,4]. Because of the reported effects of fish and LC *n*-3 PUFAs, recommendations have been made to increase oily fish and LC *n*-3 PUFA consumption for cardiovascular protection [4,13]. Oily fish intake amongst many populations is low and infrequent. An alternative source of LC *n*-3 PUFAs which can be taken regularly is supplements such as fish oil. Most fish oils contain about 30% of the active LC n-3 PUFAs eicosapentaenoic acid (EPA) and docosahexaenoic acid (DHA). Thus, a single one gram capsule of fish oil can provide about 300 mg EPA plus DHA. In most fish oils the fatty acids are found mainly as TAG. Omacor^®^ (PronovaBioPharma, Lysaker, Norway) is a highly concentrated, pharmaceutical preparation of LC *n*-3 PUFAs in ethyl ester form which contains about 84% EPA plus DHA. Omacor^®^ is able to lower plasma TAG concentrations, typically by 20% to 50% [14,15,16], and was shown in one study to lower risk of cardiovascular mortality, fatal cardiovascular events and sudden death in patients who had survived a previous myocardial infarction [17,18]. A TAG-lowering dose of Omacor^®^ is considered to be 2–4 g/day [4], while the dose used for secondary prevention of myocardial infarction was 1 g/day [17,18]. 

There have been numerous studies of LC n-3 PUFAs given as fish oil type supplements or in the form of Omacor^®^ on risk factors for CVD in a variety of patient groups including those with different risk factor profiles and at risk of different disease manifestations. There have been relatively few studies of the impact of LC *n*-3 PUFAs on CVD risk factors specifically in persons with advanced carotid atherosclerosis. We took advantage of samples from a randomised, controlled trial of Omacor^®^ given at 2 g/day to patients awaiting carotid endarterectomy [19] to assess the effects on plasma lipid and inflammatory markers concentrations. We hypothesised that Omacor^®^ would result in lower concentrations of TAG and some inflammatory markers in the plasma. 

## 2. Patients, Materials, and Methods

### 2.1. Study Design

**Figure 1:**
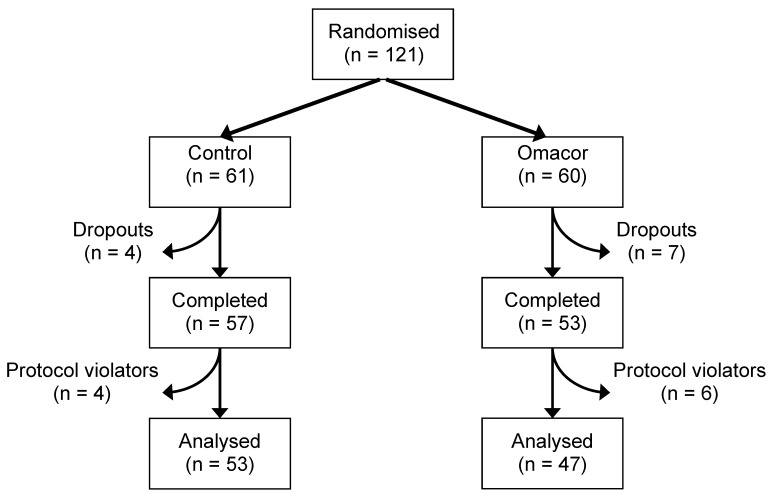
The flow of patients through the study.

Ethical permission for all procedures was obtained from the Southampton and South West Hampshire Local Research Ethics Committee and all patients gave written informed consent. The study was registered at www.clinicaltrials.gov (ClinicalTrials.gov identifier NCT00294216) and is known by the acronym OCEAN (Omacor Carotid EndArterectomy iNtervention). Patients destined to undergo carotid endarterectomy in the Southampton University Hospitals NHS Trust, Southampton or at Queen Alexandra Hospital, Portsmouth during the period March, 2003, to December, 2004, were considered eligible for entry into the study. Inclusion criteria were awaiting carotid endarterectomy, being >18 year of age and being able to give written informed consent. Exclusion criteria were inability to give written informed consent, consuming fish oil or primrose oil supplements, eating >two oily fish meals per week, being pregnant or lactating, or participating in another trial. Eligible patients were randomised in a double-blind manner to receive either olive oil capsules as placebo or LC *n*-3 PUFA ethyl esters (Omacor^®^) as capsules. Both were provided by PronovaBioPharma, Lysaker, Norway. Randomisation of patients to treatment group was according to a random number table and was performed by PronovaBioPharma. All researchers were blind to treatment allocation. Capsules were provided in sealed containers and patients took two capsules/day until surgery. The capsules were gelatine-coated and non-transparent. The amounts of EPA and DHA provided by 2 capsules of Omacor^®^ were 888 mg and 777 mg/day respectively, which is achievable in the diet with regular, but high, oily fish consumption. The amount of oleic acid provided by the olive oil placebo was 1.55 g/day; this amount is considered negligible since the typical adult consumption of oleic acid in the United Kingdom is 20–30 g/day [20]. Patients continued their usual medication throughout the study period and they were advised not to change their current diet. A total of 121 subjects were recruited and randomised; however only one hundred patients (*n* = 47 and *n* = 53 for Omacor^®^ and placebo, respectively) were used in the final analysis due to drop-out and protocol violation. Figure 1 shows the summary of the trial profile. Eleven patients withdrew from the study: four for clinical reasons, one because they could not comply with the study protocol, and six for unspecified reasons (Figure 1). A further 10 patients were excluded from the analysis study because they were identified as protocol violators, pre-defined as failure to consume more than 70% of the allocated capsules (Figure 1). Compliance was promoted by regular contact with patients and was monitored using capsule count and by analysis of the plasma fatty acid profile.

At study entry and just prior to surgery a 20 mL fasting venous blood sample was taken from the forearm into tubes containing lithium-heparin. Blood samples were collected after a 12 h overnight fast, put on ice, and plasma separated by centrifugation at 3000 rpm for 10 minutes at 4 °C. Aliquots of plasma were kept frozen at −80 °C until analysis. 

Weight and height were taken to the nearest 0.1 kg and 0.1 cm, respectively. Body mass index (BMI) was calculated as weight (in kg) divided by the square of the standing height (m^2^). Two-blood pressure measurements were obtained (Marquette^®^; San Juan, CA, USA) in the supine position from the non-dominant side arm with an additional reading was taken if values from two consecutive measurements were more than 10 mm Hg apart. The mean of the two measurements was used.

### 2.2. Measurement of Plasma Lipid Concentrations

Plasma TAG, total cholesterol, and high density lipoprotein (HDL) cholesterol concentrations were measured using commercial kits from Konelab™ (Vantaa, Finland) and a Konelab™ auto-analyser. Low density lipoprotein (LDL) cholesterol was calculated using the Fridewald equation.

### 2.3. Measurement of Plasma Inflammatory Marker Concentrations

Plasma concentrations of interleukin (IL)-6, IL-10, soluble endothelial (sE)-selectin, soluble intercellular adhesion molecule (sICAM)-1, soluble vascular cell adhesion molecule (sVCAM)-1, matrix metalloproteinase (MMP)-2, MMP-9, C-reactive protein (CRP), transforming growth factor (TGF)-β1, soluble CD40 ligand (sCD40L), interferon gamma induced protein (IP)-10, and monokine-induced by gamma-interferon (MIG) were measured using commercial ELISA kits. sE-selectin, sICAM-1 and sVCAM-1 kits were from Biosource Europe, Nivelles, Belgium; IL-6, IL-10, MMP-2, MMP-9, sCD40L, IP-10, MIG, and TGF-β1 kits were from R&D Systems (Minneapolis, MN, USA); high sensitivity CRP kits were from Diagnostic System Laboratories (Webster, TX, USA). For all assays, the manufacturer’s instructions were followed and the absorbance was read on a plate reader using 450 nm as the primary wavelength and 610–650 nm as the reference wavelength. The sensitivity of each assays was: <0.039 pg/mL (IL-6), <0.5 ng/mL (IL-10), 0.5 ng/mL (sE-selectin), <0.5 ng/mL (sICAM-1), 0.9 ng/mL (sVCAM-1), 0.16 ng/mL (MMP-2), <0.156 ng/mL (MMP-9), <4.61 pg/mL (TGF-β1), <10.1 pg/mL (sCD40L), <4.46 pg/mL (IP-10), <11.3 pg/mL (MIG), and 1.6 ng/mL (CRP). 

### 2.4. Statistical Analysis

The Kolmogorov-Smirnov and Shapiro-Wilk tests were applied to assess normality of data. Data for continuous variables that were normally distributed are presented as mean values and their standard deviations (SD) whilst non-normally distributed data are presented as medians and 10th and 90th percentiles. Comparison of normally distributed data between groups was performed using the independent *t*-test and within a group using the paired Student’s *t*-test. Non-normally distributed data were compared using the Wilcoxon signed ranks and Mann-Whitney U tests. Relationships between variables were evaluated using Pearson’s correlation coefficient or Spearman’s rank correlation. In all cases a value for *p* ≤ 0.05 was taken to indicate a significant effect. SPSS version 14.02 (SPSS Inc., Chicago, IL, USA) was used for all statistical analyses.

## 3. Results

### 3.1. Characteristics of the Patients

**Figure 2:**
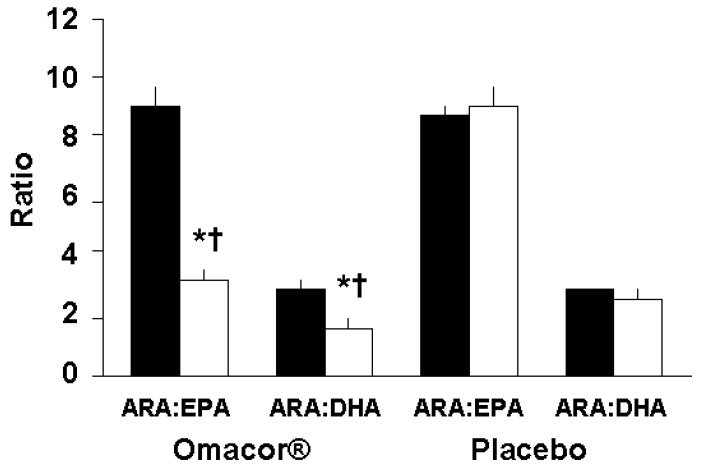
Summary of fatty acid composition changes in plasma phosphatidylcholine in the Omacor^®^ and placebo groups Bars are mean ± SEM. Black bars represent study entry; open bars represent end of supplementation period. ARA, arachidonic acid; DHA, docosahexaenoic acid; EPA, eicosapentaenoic acid. * Indicates significantly different from study entry (*p* < 0.001). ^†^ Indicates significantly different from placebo (*p* < 0.001).

Characteristics of the 100 patients, included here, including blood pressure, and blood lipid and inflammatory marker concentrations, were not significantly different between the groups at baseline (Table 1). The median durations of supplementation were 21 (8–57; 10th–90th percentile) and 22 (9–66; 10th–90th percentiles) days for the Omacor^®^ and placebo groups, respectively, and ranged between 7 and 102 days. Based on the counting of the returned capsules, compliance was high (95.5% and 95.1% for Omacor^®^ and placebo groups, respectively) and did not differ between groups (*p* = 0.808). Plasma phosphatidylcholine fatty acid composition was reported previously [19] and showed a significant increase in EPA (from 1.3% ± 0.6% to 3.3% ± 0.9% of total fatty acids), docosapentaenoic acid (from 1.0% ± 0.3% to 1.3% ± 0.3% of total fatty acids) and DHA (from 3.7% ± 1.3% to 5.8% ± 1.2% of total fatty acids) in the Omacor^®^ group (all *p* < 0.001 *vs.* baseline) such that these fatty acids were higher in the Omacor^®^ group than in the placebo group at the end of supplementation (all *p* < 0.005). The proportion of ARA was not significantly altered by Omacor^®^ [19]. As a result of the increases in EPA and DHA content in plasma phosphatidylcholine in the Omacor^®^ group, the ratios of ARA to EPA and of ARA to DHA were significantly decreased (both *p* < 0.001 *vs.* baseline; Figure 2) and were lower in the Omacor^®^ group than in the placebo group at the end of supplementation (both *p* < 0.001; Figure 2). There were no significant changes in fatty acids in the placebo group [19]. 

**Table 1:** Baseline characteristics of the patients in the Omacor^®^ and placebo groups.

	Omacor^®^ (*n* = 47)	Placebo (*n* = 53)
Sex (*n*)		
Male	32	36
Female	15	17
Smoking status (*n*)		
Yes	8	8
No	8	11
Ex-smokers	31	34
Medication use (*n*)		
Aspirin	41	38
Anti-coagulant	13	5
Beta-blocker	17	16
Calcium channel blocker	18	16
ACE-inhibitors	28	27
Statin	45	39
Diuretics	26	25
Nitrates	13	8
Oral hypoglycaemic agents	10	13
Insulin	2	1
Age (year)	72.0 (10.7)	73.0 (8.3)
BMI (kg/m^2^)	27.1 (4.9)	26.5 (3.7)
Systolic blood pressure (mm Hg)	155.3 (27.9)	155.2 (22.1)
Diastolic blood pressure (mm Hg)	80.6 (13.9)	82.0 (13.3)
Total cholesterol (mmol/L)	4.8 (1.1)	4.9 (1.2)
LDL-cholesterol (mmol/L)	2.5 (1.6–4.3)	2.7 (1.5–4.4)
HDL-cholesterol (mmol/L)	1.3 (0.9–2.4)	1.2 (0.9–1.9)
Triglycerides (mmol/L)	1.3 (0.7–2.2)	1.3 (0.7–2.6)
Total cholesterol:HDL-cholesterol ratio	3.2 (2.4–4.8)	3.8 (2.6–5.0)
LDL-cholesterol:HDL-cholesterol ratio	1.8 (1.0–3.6)	2.2 (1.3–3.2)
sICAM-1 (ng/mL)	167 (73–426)	216 (65–445)
sVCAM-1(ng/mL)	673 (226–1578)	594 (272–1131)
sE-selectin (ng/mL)	92.0 (33.0–234.4)	91.4 (15.6–290.0)
IL-6 (pg/mL)	1.2 (0.4–4.0)	1.2 (0.1–4.8)
IL-10 (pg/mL)	1.5 (0–5.24)	0.5 (0–2.9)
MMP-2 (ng/mL)	192 (129-290)	191 (129–294)
MMP-9 (ng/mL)	167 (47–421)	138 (29–389)
TGF-β1 (ng/mL)	9308 (2394–19170)	9788 (3356–16844)
CRP (mg/L)	1.0 (1.0–31.7)	1.0 (1.0–9.1)
sCD40L (pg/mL)	776 (243–2239)	774 (213–3140)
IP-10 (pg/mL)	103.9 (51.6–273.3)	102.8 (47.8–289.4)
MIG (pg/mL)	119.3 (37.9–360.7)	107.4 (30.6–294.3)

Data are mean (SD) or median (10th–90th percentile); ACE, angiotensin-converting-enzyme; BMI, Body mass index; LDL, low density lipoprotein; HDL, high density lipoprotein; sICAM, soluble intercellular adhesion molecule; sVCAM, soluble vascular cellular adhesion molecule; sE, soluble endothelial; IL, interleukin; MMP, matrix metalloproteinase; TGF, transforming growth factor; CRP, C-reactive protein, sCD40L, soluble CD40 ligand; IP, interferon gamma induced protein; MIG, monokine induced by gamma-interferon.

### 3.2. Effect of Supplementation on BMI, Blood Pressure, and Plasma Lipid Profile

Table 2 shows the BMI, blood pressure and plasma lipid concentrations at baseline and after supplementation with Omacor^®^ or placebo. There were significant reductions in both systolic and diastolic blood pressure and in plasma TAG, total cholesterol, and LDL-cholesterol concentrations in both groups (Table 2). There was an average 20% reduction in plasma TAG concentration in the Omacor^®^ group; the average reduction in the placebo group was less than half of this. There were no significant effects on total cholesterol:HDL-cholesterol and LDL-cholesterol:HDL-cholesterol ratios in either group (Table 2). At the end of the supplementation period, there were no differences in blood pressure or plasma lipid concentrations between the two groups.

### 3.3. Effect of Supplementation on Plasma Inflammatory Markers

The concentrations of plasma inflammatory markers at baseline and after supplementation with Omacor^®^ or placebo are shown in Table 3. sE-selectin, sVCAM-1 and MMP-2 concentrations were significantly decreased in the Omacor^®^ group (Table 3). However, decreases in sVCAM-1 and MMP-2 also occurred in the placebo group (Table 3). sE-selectin concentration increased in the placebo group (Table 3). None of the other inflammatory markers was affected by either Omacor^®^ or placebo and, at the end of supplementation, none of the inflammatory markers differed between the two groups (Table 3). 

**Table 2:** Blood pressure and plasma lipid concentrations at baseline and after supplementation with Omacor^®^ or placebo.

	Omacor^®^ (*n* = 47)			Placebo (*n* = 53)	
	Before	After	Baseline		After
BMI (kg/m^2^)	27.1 (4.9)	27.0 (4.8)	26.5 (3.7)		26.3 (3.9)
SBP (mm Hg)	155.3 (27.9)	142.7 * (23.6)	155.2 (22.1)		142.0 ** (19.2)
DBP (mm Hg)	80.6 (13.9)	72.4 ** (10.2)	82.0 (13.3)		73.8 ** (11.3)
TAG (mmol/L)	1.31 (0.70–2.22)	1.00 *** (0.56–1.62)	1.30 (0.74–2.60)		1.10 ** (0.60–2.40)
Total cholesterol (mmol/L)	4.8 (0.2)	4.3 ** (0.2)	4.9 (0.2)		4.3 ** (0.2)
HDL-cholesterol (mmol/L)	1.31 (0.93–2.41)	1.25 *** (0.85–2.06)	1.23 (0.88–1.88)		1.07 *** (0.70–1.77)
LDL-cholesterol (mmol/L)	2.50 (1.62–4.28)	2.46 * (1.56–3.67)	2.68 (1.50–4.37)		2.50 * (1.47–3.89)
Total:HDL-cholesterol ratio	3.2 (2.4–4.8)	3.4 (2.5–5.1)	3.8 (2.6–5.0)		3.9 (2.4–5.4)
LDL-cholesterol:HDL-cholesterol	1.8 (1.0–3.6)	2.0 (1.2–3.4)	2.2 (1.3–3.2)		2.4 (1.3–3.7)

Data are mean (SD) or median (10th–90th percentile); * Significantly different from baseline (*p* < 0.05); ** Significantly different from baseline (*p* < 0.01); *** Significantly different from baseline (*p* < 0.001); BMI, body mass index; SBP, systolic blood pressure; DBP, diastolic blood pressure; TAG, triglycerides; HDL, high density lipoprotein; LDL, low density lipoprotein.

**Table 3:** Plasma inflammatory markers before and after supplementation with Omacor^®^ or placebo.

	Omacor^®^ (*n* = 47)		Placebo (*n* = 53)	
	Baseline	After	Baseline	After
CRP (mg/L)	1.0 (1.0–31.7)	1.0 (1.0–20.0)	1.0 (1.0–9.1)	1.0 (1.0–23.0)
sE*-*selectin (ng/mL)	92.0 (33.0–234.4)	60.3 ** (17.4-240.7)	91.4 (15.6–290.0)	95.4 * (16.4–274.9)
sICAM-1 (ng/mL)	167.1 (73.2–425.7)	146.3 (69.2–383.3)	216.1 (64.9–444.7)	202.0 (74.2–345.5)
sVCAM-1 (ng/mL)	673 (226–1578)	544 *** (252–1146)	594 (272–1131)	489 ** (236–933)
IL-6 (pg/mL)	1.2 (0.2–4.0)	1.0 (0.2–4.1)	1.2 (0.1–4.8)	0.9 (0–3.7)
IL-10 (pg/mL)	1.5 (0–5.2)	0.9 (0–6.2)	0.5 (0–2.9)	0.8 (0–3.5)
MMP-2 (ng/mL)	192.2 (129.2–290.3)	165.7 *** (100.8–248.4)	191.2 (129.0–293.7)	155.3 *** (71.8–233.2)
MMP-9 (ng/mL)	167.2 (46.6–421.1)	163.3 (32.0–558.3)	138.0 (28.8–389.3)	152.1 (34.2–403.2)
TGF-β1 (ng/mL)	9308 (2394–19170)	7908 (2956–13690)	9788 (3356–16844)	7516 (3484–17388)
sCD40-L (ng/mL)	776 (243–2239)	655 (179–1677)	774 (193–2906)	659 (248–2285)
IP-10 (pg/mL)	103.9 (51.6–273.3)	100.2 (55.4–353.1)	102.8 (47.8–289.4)	114.9 (35.9–295.9)
MIG (pg/mL)	119.3 (37.9–360.7)	107.2 (30.6–320.6)	107.4 (30.6–294.3)	97.4 (16.9–327.2)

Data are median (10th–90th percentile); * Significantly different from baseline (*p* < 0.05); ** Significantly different from baseline (*p* < 0.01); *** Significantly different from baseline (*p* < 0.001); CRP, C-reactive protein; sE, soluble endothelial; sICAM, soluble intercellular adhesion molecule; sVCAM, soluble vascular cellular adhesion molecule; IL, interleukin; MMP, matrix metalloproteinase; TGF, transforming growth factor; sCD40L, soluble CD40 ligand; IP, interferon gamma induced; protein 10; MIG, monokine induced by gamma interferon.

## 4. Discussion

This double-blind, placebo-controlled study of the effect of moderate dose (2 g/day) Omacor^®^ (providing 1.665 g/day of EPA plus DHA) was carried out in patients awaiting carotid endarterectomy, a fairly unexplored group as far as the study of LC *n*-3 PUFAs is concerned. We previously reported the effects of Omacor^®^ on plasma phosphatidylcholine and atherosclerotic plaque fatty acid composition and on plaque characteristics and inflammatory gene expression in these patients [19]. The dose of Omacor^®^ used is at the bottom of the TAG lowering range [4], although Omacor^®^ was used at 1 g/day in the GISSI trial, where it significantly reduced overall mortality, cardiovascular mortality, and sudden death [17,18]. LC *n*-3 PUFAs have been shown to lower blood pressure [7,8] and to reduce inflammation [11,12]. The current study identified a reduction in plasma TAG concentrations, blood pressure and some of the inflammatory markers in the Omacor^®^ group. However, in most cases similar changes also occurred in the placebo group. The reason for this is not clear, but it is possible that the patients made some lifestyle changes induced by the knowledge that they would be going to surgery for their condition. The one difference between the effects of Omacor^®^ and the placebo was in the concentration of sE-selectin, which decreased with Omacor^®^ but increased in the placebo group.

It has been reported that LC *n*-3 PUFAs can increase total cholesterol concentration by 5% to 10% and decrease TAG concentration by 20% to 50% [9,21,22]. In the current study total cholesterol concentration decreased by 13% in the Omacor^®^ group compared with 9% in the placebo group. TAG concentrations were decreased by 20% in the Omacor^®^ group compared to 9% in the placebo group. This indicates that compared with placebo Omacor^®^ lowered fasting plasma TAG by an average of 11%. It is also important to note that most patients were already taking medication to control blood lipids, particularly statins. Omacor^®^ is indicated as an adjunct to diet to lower high plasma TAG concentrations in adult patients and has been shown to be effective in lowering plasma TAG concentrations when used in combination with statins [23]. The combination of Omacor^®^ plus simvastatin improved lipoprotein parameters to a greater extent than simvastatin alone [23]. Although statins have great utility as cholesterol-lowering agents, statin therapy is reported to result in unfavourable changes in plasma fatty acids with an increase in the ratios of ARA to EPA and ARA to DHA ratios [24], which is considered to be deleterious. In the current study Omacor^®^ lowered these ratios. Although fairly similar amounts of EPA and DHA were provided in the Omacor^®^ group, the ratio of ARA to EPA was decreased by an average of 65%, while the ratio of ARA to DHA was decreased by an average of 50%. This reflects the greater ease of incorporation of EPA into phospholipids. 

In the current study sE-selectin and sVCAM-1 concentrations decreased by about 25% and MMP-2 concentration by about 12% in the Omacor^®^ group. None of the other inflammatory markers measured were affected by Omacor^®^. There are reports that EPA and DHA can reduce production of several inflammatory cytokines *in vitro* and that high dose LC *n*-3 PUFAs decrease *ex vivo* production of TNF, IL-1β, and IL-6 especially in healthy volunteers [11] and lower the concentrations of sICAM-1 [25] and sVCAM-1 [26]. However, lower doses of LC *n*-3 PUFAs seem to be ineffective towards cytokine production [11] and the reported effects on soluble adhesion molecule concentrations are highly variable [14,25,26,27,28]. Likewise, effects of LC *n*-3 PUFAs on CRP concentrations are not consistent [29], although some studies have reported a decrease in CRP concentration with a nutritional formula providing a low dose of LC *n*-3 PUFAs [30]. Low dose Omacor^®^ (1 g/day) did not influence plasma IL-6 concentration in patients studied following myocardial infarction [31], while others reported a lack of effect of Omacor^®^ on sCD40L and MMP-9 concentrations [32]. 

In conclusion, the current study found a modest TAG lowering effect of 2 g/day Omacor^®^ compared with placebo and identified that one inflammatory marker, sE-selectin, is affected by this dose of Omacor^®^. The most likely explanations for these limited effects of Omacor^®^ are use of medications to control blood lipids, blood pressure, and inflammation by the patients studied; the fairly low starting plasma TAG concentrations; the low dose of LC *n*-3 PUFAs provided (1.665 g/day) which is at the bottom end of the specified TAG lowering dose and may be below the doses needed for significant impact on blood pressure and inflammation; and the short duration of the intervention (median 21 days).

## 5. Conclusions

Omacor^®^ given at 2 g/day for an average of 21 days to patients with advanced carotid atherosclerosis lowers triglycerides and soluble E-selectin concentrations, but has limited broad impact on the plasma lipid profile or on inflammatory markers. This may be because the duration of intervention was too short or the dose of *n*-3 fatty acids was too low.
